# The Swedish Web Version of the Quality of Recovery Scale Adapted for Use in a Mobile App: Prospective Psychometric Evaluation Study

**DOI:** 10.2196/mhealth.9061

**Published:** 2017-12-03

**Authors:** Ulrica Nilsson, Karuna Dahlberg, Maria Jaensson

**Affiliations:** ^1^ School of Health Sciences Faculty of Medicine and Health Örebro University Örebro Sweden

**Keywords:** psychometric evaluation, postoperative recovery, Web version, evaluation studies, mobile application, Quality of Recovery scale

## Abstract

**Background:**

The 40-item Quality of Recovery (QoR-40) questionnaire is well validated for measuring self-assessed postoperative recovery. The Swedish version of the 40-item Quality of Recovery (QoR-40) has been developed into a Web-based questionnaire, the Swedish Web version of the Quality of Recovery (SwQoR) questionnaire, adapted for use in a mobile app, Recovery Assessment by Phone Points, or RAPP.

**Objective:**

The aim of this study was to test the validity, reliability, responsiveness, and clinical acceptability and feasibility of SwQoR.

**Methods:**

We conducted a prospective psychometric evaluation study including 494 patients aged ≥18 years undergoing day surgery at 4 different day-surgery departments in Sweden. SwQoR was completed daily on postoperative days 1 to 14.

**Results:**

All a priori hypotheses were confirmed, supporting convergent validity. There was excellent internal consistency (Cronbach alpha range .91-.93), split-half reliability (coefficient range .87-.93), and stability (*ri*=.99, 95% CI .96-.99; *P*<.001). Cohen *d* effect size was 1.00, with a standardized response mean of 1.2 and a percentage change from baseline of 59.1%. An exploratory factor analysis found 5 components explaining 57.8% of the total variance. We noted a floor effect only on postoperative day 14; we found no ceiling effect.

**Conclusions:**

SwQoR is valid, has excellent reliability and high responsiveness, and is clinically feasible for the systematic follow-up of patients’ postoperative recovery.

## Introduction

Day surgery (ie, minor surgery) is an expanding, well-established practice internationally. Surgical and anesthetic advances, in day surgery in particular, have drastically reduced mortality and major morbidity frequencies [1]. Before discharge, patients admitted for day surgery are monitored postoperatively for only a few hours; they must then assume primary responsibility for managing their own recovery [2,3]. There are numerous postdischarge symptoms, such as pain, drowsiness, fatigue and tiredness, postoperative nausea and vomiting, sleep disturbance, and sore throat [4-7]. Although such symptoms are rarely life threatening, they may be unpleasant and disturbing, extending the recovery time and delaying resumption of normal activity [2,8]. The introduction of patient-centered care has made recovery a multidimensional construct, and recovery assessment tools address physical (nociceptive), functional (activities of daily living), cognitive, and psychological (emotive, satisfaction) outcomes [8].

The 40-item Quality of Recovery (QoR-40) questionnaire is well validated for measuring self-assessed postoperative recovery [9,10]. This questionnaire had been previously tested in a population of Swedish day-surgery patients [11]. A meta-analysis that included the Idvall et al study demonstrated the QoR-40’s high validity, reliability, responsiveness, and clinical utility in a broad range of patient populations [7]. However, all the included studies relied on paper-based assessments during postoperative recovery. Instead of paper-based postoperative follow-up, the use of mobile phones could be ideal, as many people of all ages and across socioeconomic and geographic boundaries own these ubiquitous devices. The Swedish version of QoR-40 has therefore been further developed into a Web-based instrument, the Swedish Web version of the Quality of Recovery (SwQoR) questionnaire, and adapted for use in the Recovery Assessment by Phone Points (RAPP) mobile app [12-14] equivalent to the paper version of the SwQoR [14].

SwQoR is a multi-item questionnaire including 24 negatively worded items rated on 11-point visual analog scales (VASs) ranging from 0, “none of the time,” to 10, “all of the time” [15]. That all items are negatively worded differs from the QoR-40, which includes both positive and negative items. In an earlier study by our research group [14], patients reported that, as they respond to the items one by one, they would find it easier if all of the items were either positively or negatively worded. As patients undergoing surgery are used to rating their postoperative pain using a VAS or numeric rating scale, and most of the items (n=17) were negatively worded, all positive items (n=7) were reformulated into negatively worded items [13,15]. To facilitate responding to each item, a dot on the VAS line is programmed to return to a score of 5 each time a new item appears on the screen, clarifying that a new item is to be responded to [12]. Each item appears separately on the screen and the dot must be moved to indicate a response. The item disappears from the screen immediately after a response is given, and each item must be responded to before the patient can submit the daily assessment [15]. The global score for SwQoR ranges from 0 to 240, with good postoperative recovery indicated by a score of 0 to 31 and poor postoperative recovery indicated by a score of 32 or more (ie, more discomfort) on postoperative day 7 [16].

The aim of this study was to undertake a psychometric evaluation of SwQoR in a day-surgery population.

## Methods

This psychometric evaluation study is a part of a multicenter, 2-group, parallel, single-blind randomized controlled trial with the primary aim to estimate the cost effectiveness of using, versus not using, RAPP for follow-up on recovery after day surgery (trial registration NCT02492191 [15]). The study was conducted from October 2015 to July 2016 at 4 day-surgery departments in Sweden. Here we present data only on participants who were randomly allocated into the intervention group. Study implementation upheld the ethical standards of the Declaration of Helsinki (6th revision) and was approved by the Uppsala/Örebro Regional Ethics Committee (2015/262).

### Sample and Procedure

Patients were told of the study and invited to enroll on the day of surgery. Written information about the study was also sent out in advance, together with information about the planned surgery. Oral information was provided preoperatively on the day of surgery, and all participants gave oral and written consent. The research nurse responsible for participant recruitment at the day-surgery department ensured that all participants eligible for study participation were invited to enroll. Inclusion criteria were undergoing day surgery, over 17 years of age, access to a mobile phone, and able to understand written and spoken Swedish. Exclusion criteria were visual impairment, memory impairment, substance abuse, or undergoing a surgical abortion.

Preoperatively, the research nurse installed RAPP, including SwQoR, on each participant’s own mobile phone. The participants were individually briefed and allowed to test the app by inputting sample responses. The research nurse explained in detail the RAPP functionalities, such as how to move between items, input responses, and use the navigation keys. The participants completed SwQoR daily for 14 days using RAPP and received a daily reminder via the app.

Preoperatively, we measured overall health using the paper-based EuroQol visual analog scale (EQ VAS), comprising a vertically graduated scale ranging from 0, “worst imaginable health state,” to 100, “best imaginable health state” [17].

We gathered participants’ demographic and pre- and postoperative data from their patient records, which included age, sex, American Society of Anesthesiologists (ASA) physical status classification, type of anesthesia, and duration of postoperative stay calculated from when the patient entered the postanesthesia care unit (PACU) to the time of discharge.

### Psychometric Evaluation

The psychometric evaluation was guided by the Consensus-Based Standards for the Selection of Health Measurement Instruments [18] and previous psychometric evaluations of the QoR-40 [9,10,19] and QoR-15 questionnaires [20,21].

#### Acceptability and Feasibility

We assessed *acceptability* and *feasibility*, which measure clinical user friendliness, in terms of (1) participant recruitment rate, days 1 to 14; and (2) successful response rate, days 1 to 14.

#### Floor or Ceiling Effects

We deemed floor or ceiling effects to be present if over 15% of participants reported, respectively, the highest or lowest postoperative SwQoR score on days 1 to 14 [22].

#### Validity

We assessed *validity*, which evaluates the accuracy, in terms of *construct validity* and *discriminant validity*.

*Construct validity* is the extent to which questionnaire scores are consistent with hypotheses, assuming that the questionnaire validly measures the construct addressed. We assumed a correlation coefficient of .3< *r*<.7 to indicate moderate correlation. To analyze construct validity, we conducted a priori hypothesis testing, hypothesizing that SwQoR on day 14 would correlate negatively with EQ VAS on day 14 postoperatively: that is, high scores of SwQoR (ie, poor quality of recovery) correlate with low quality of life. We expected lower correlations (ie, ρ<.3) due to day surgery between SwQoR on day 1 postoperatively and duration of surgery, duration of PACU stay, and age. In addition, we expected higher scores of SwQoR (ie, poor quality of recovery) in female versus male patients and in general anesthesia versus regional anesthesia.

*Discriminant validity*, tested on days 1 to 7 and 14, suggested that patients with low overall health as defined by an EQ VAS score of <76 mm preoperatively (guided by the mean value of 75 in this study) would have higher scores on SwQoR (ie, poor postoperative recovery).

#### Reliability

We assessed *reliability*, which evaluates the consistency of results, in terms of the following 4 measures.

*Internal consistency* was measured as the average correlation between the SwQoR items on days 1 to 14, indicated by Cronbach alpha, as well as between the items captured by the factors emerging in the exploratory factor analysis (EFA).

*Split-half reliability* was measured by the correlation between randomly split segments of SwQoR on days 1 to 14.

*Exploratory factor analysis* identified the underlying relationships between the 24 items on day 1.

*Test-retest reliability* was assessed by having a subset of patients (n=17, mean age 48.8 years, 8 male and 9 female patients, 9 ASA I and 8 ASA II) complete SwQoR twice on one of postoperative days 1 to 7 within a time frame of 2 to 30 minutes (mean 6 minutes); we then assessed the correlation between the repeated questionnaire results.

#### Responsiveness

We assessed *responsiveness*, which evaluates SwQoR’s sensitivity and ability to detect clinically important changes, in terms of the following 3 measures.

*Cohen* d *effect size* was calculated as the average changes in scores from days 1 to 7, 1 to 14, and 7 to 14, divided by the pooled standard deviation of all measurements: 0.2 to 0.5 indicates a small effect, 0.5 to 0.8 indicates a moderate effect, 0.8 to 1.2 indicates a large effect, 1.2 to 2.0 indicates a very large effect, and >2.0 indicates a huge effect [23].

*Standardized response mean* (SRM) was calculated as the mean change in scores divided by the standard deviation of this change, with values of 0.20, 0.50, and 0.80 or greater being considered small, moderate, and large effect sizes, respectively [24].

*Mean changes over time* and *percentage changes from baseline* from days 1 to 7, 1 to 14, and 7 to 14 were calculated.

### Statistical Analysis

We present data as mean (SD), numbers, percentages, ranges, or 95% CI for the sake of clarity. Although the questionnaire is considered to be an ordinal scale, the data were skewed and we performed nonparametric tests. All percentages are rounded up to the nearest integer. We measured associations using Spearman rank correlation coefficients (ρ). We assessed internal consistency with Cronbach alpha. To detect differences between sex and type of anesthesia, we performed Mann-Whitney *U* tests. We assessed test-retest reliability with the intraclass correlation coefficient (*ri*). We used IBM SPSS version 24 (IBM Corporation) for Windows for the statistical analyses. We rejected the null hypothesis if the 2-tailed *P*<.05.

## Results

### Acceptability and Feasibility

In the main study, 1796 patients were eligible for inclusion. Of these, 433 did not meet the inclusion criteria and 336 declined to participate, resulting in 1027 day-surgery patients who were included for random allocation. Of the 513 patients randomly allocated to the intervention group, we excluded 19 due to canceled operations (n=15), refusal to participate (n=3), and technical issues (n=1). Thus, 494 patients were covered in this study dataset. Table 1 presents patients’ demographic variables and perioperative factors.

The response rate was 86.8% (n=429) on postoperative day 1, then 69.0% (n=341) on day 7, and 57.5% (n=284) on day 14. The global SwQoR score decreased from 49.3 (SD 34.2) on day 1 to 19.5 (SD 25.0) on day 14 (Table 2). There were no missing items because each item had to be responded to before submitting the daily assessment.

### Floor or Ceiling Effects

The distributions of SwQoR global scores on days 1, 7, and 14 were skewed to the left and ranged from 0 to 191, from 0 to 178, and from 0 to 133, respectively. We found a floor effect on day 14, when 45 (15.8%) participants reported SwQoR global scores of 0. No ceiling effects were present (Table 2).

### Validity

*Construct validity* analysis indicated a moderate and negative correlation between the SwQoR and EQ VAS results on postoperative day 14 (ρ=–.53, *P*<.001). As hypothesized, the construct validity results indicated rather low correlations between SwQoR and PACU stay (ρ=.28, *P*<.001), duration of surgery (ρ=.17, *P*<.001), and age (ρ=.20, *P*<.001). Women reported significantly higher mean SwQoR scores (ie, poorer recovery) than men (mean 53.2, SD 35.7 vs 46.3, SD 33.8; *P*=.04). These differences were also significant between general (mean 52.7, SD 35.5) and regional anesthesia (mean 39.3, SD 27.1; *P*=.02). All hypotheses were therefore confirmed.

We determined *discriminant validity* by comparing patients with good versus poor overall health, as defined by EQ VAS scores of ≥75 or <75 mm, respectively. The lower SwQoR scores on day 1 of those patients with good overall health indicated significantly better recovery (mean 44.3, SD 32.4 vs 59.4, SD 35.5 for patients with poor health), for a mean difference of 15.1 (95% CI 8.1-22.0; *P*<.001).

**Table 1:** Participants’ demographic variables, and surgical and anesthetic factors (n=494).

Variables		Data
**Sex n (%)**		
	Male	220 (44.5)
	Female	274 (55.5)
**Age (years)**		
	Mean (SD^a^)	45 (15)
	Median (range)	46 (18-81)
**ASA**^b^ **classification, n (%)**		
	I	242 (49.0)
	II	147 (29.8)
	III	11 (2.2)
	Missing information	84 (17.0)
**Type of anesthesia, n (%)**		
	General anesthesia	362 (73.3)
	Regional or local anesthesia	107 (21.7)
	Missing information	25 (5.1)
**Type of airway management, n (%)**		
	Endotracheal tube	77 (15.6)
	Laryngeal mask airway	267 (54.1)
	Mask ventilation	6 (1.2)
	Spontaneous breathing	119 (24.1)
	Missing information	25 (5.1)
**Type of surgery, n (%)**		
	Orthopedic	160 (32.4)
	General	126 (25.5)
	Hand	116 (23.5)
	Ear, nose, and throat	52 (10.5)
	Gynecological	26 (5.3)
	Eye	5 (1.0)
	Urological	3 (0.6)
	Dental	2 (0.4)
Duration of surgery (minutes), mean (SD)		40 (29.6)
PACU^c^ stay (minutes), mean (SD)		151 (63.0)

^a^SD: standard deviation.

^b^ASA: American Society of Anesthesiologists.

^c^PACU: postanesthesia care unit.

**Table 2:** SwQoR^a^ response rate, mean and range, floor effect, Cronbach alpha, and split-half coefficient on days 1-14 (n=494).

Postoperative day	Response rate, n (%)	SwQoR			Cronbach alpha	Split-half coefficient
		Mean (SD^b^)	Range	Floor effect^c^, n (%)		
1	429 (86.8)	49.3 (34.2)	0-191	3 (0.7)	.91	.87
2	405 (82.0)	42.3 (34.2)	0-201	6 (1.5)	.92	.88
3	393 (82.7)	35.2 (32.3)	0-179	12 (3.1)	.93	.91
4	380 (80.2)	34.5 (32.5)	0-177	11 (2.9)	.93	.91
5	363 (73.5)	29.7 (30.8)	0-169	13 (3.6)	.93	.91
6	356 (72.1)	26.7 (27.2)	0-155	17 (4.8)	.93	.90
7	341 (69.0)	29.4 (30.4)	0-178	15 (4.4)	.93	.91
8	336 (68.0)	26.6 (29.7)	0-172	21 (6.3)	.93	.88
9	325 (66.0)	24.5 (27.9)	0-174	26 (8.0)	.92	.93
10	310 (62.8)	22.1 (25.8)	0-133	25 (8.1)	.92	.88
11	305 (61.7)	22.7 (26.8)	0-140	25 (8.2)	.92	.89
12	304 (61.5)	21.3 (26.6)	0-128	32 (10.5)	.93	.89
13	320 (64.8)	18.8 (25.0)	0-131	46 (14.4)	.93	.87
14	284 (57.5)	17.5 (22.5)	0-133	45 (15.8)	.92	.91

^a^SwQoR: Swedish Web version of the Quality of Recovery.

^b^SD: standard deviation.

^c^Participants scoring 0.

These differences were also significant on day 7 (mean 23.7, SD 24.8 vs 40.3, SD 36.6; mean difference 16.6, 95% CI 10.1-23.2; *P*<.001), and on day 14 (13.1, SD 16.6 vs 27.1, SD 29.1; mean difference 14.0, 95% CI 8.6-20.6; *P*<.001).

### Reliability

*Internal consistency*, indicated by Cronbach alpha, ranged from .91 to .93, while the split-half coefficient ranged from .87 to .93 (Table 2). Test-retest reliability was indicated by an intraclass correlation coefficient of *ri*=.99 (95% CI .96-.99; *P*<.001).

For EFA, in assessing sampling adequacy, the Kaiser-Meyer-Olkin test result exceeded .70, with a value of .91, and Bartlett test of sphericity indicated a significant result (χ^2^_276_=4393, *P*<.001). These measures allowed us to legitimately perform EFA. The EFA gave a 5-factor solution and no forcing was necessary. The eigenvalue of the factor explaining most of the observed variance was 8.1. The EFA of the 24 items found factor loadings of .34 to .81, with 5 components identified as explaining 57.8% of the total variance. Cronbach alpha ranged from .74 to .88 for 4 of the factors and was .43 for 1 factor (Table 3).

### Responsiveness

We found Cohen *d* effect sizes of 0.62 and 1.00 and SRMs of 0.82 and 1.20 between days 1 and 7 and between days 1 and 14, respectively. The mean change from baseline was –29.15 (range –25.98 to 32.32) between days 1 and 14 (Table 4).

**Table 3:** Item mean scores and factor loadings of SwQoR^a^ day 1, by exploratory factor analysis and Cronbach alpha.

Item	Item score, mean (SD^b^)	Component				
		1	2	3	4	5
Cronbach alpha		.88	.75	.75	.74	.43
Sleeping difficulties	3.04 (3.02)	.62				
Not having a general feeling of well-being	3.10 (2.08)	.78				
Not feeling in control of my situation	2.49 (2.90)	.59				
Having difficulty feeling relaxed or comfortable	3.07 (2.86)	.80				
Depressed	1.71 (2.35)	.66				
Anxious	2.00 (2.40)	.62				
Difficulties concentrating	2.09 (2.58)	.55				
Fever	0.56 (1.40)		.50			
Voice not sounding the same as usual	1.44 (2.56)		.67			
Sore throat	1.62 (2.61)		.79			
Sore mouth	0.77 (1.87)		.79			
Having trouble breathing	0.68 (1.69)		.67			
Having difficulty taking care of my personal hygiene	2.54 (2.75)			.62		
Having difficulty returning to work or usual home activities	6.28 (3.40)			.58		
Nausea, vomiting, or both	1.59 (2.52)			.64		
Dizziness	2.03 (2.64)			.71		
Headache	1.68 (2.49)			.52		
Muscle pain	2.94 (2.69)				.43	
Pain in the surgical wound	5.02 (3.09)				.57	
Reddened surgical wound	1.66 (2.52)				.77	
Swollen surgical wound	2.58 (3.11)				.81	
Trouble urinating	0.83 (1.84)					.55
Diarrhea	0.35 (1.12)					.66
Feeling constipated	1.01 (2.15)					.34

^a^SwQoR: Swedish Web version of the Quality of Recovery.

^b^SD: standard deviation.

**Table 4:** Cohen *d* effect size, mean change, and standardized response mean, days 1-7, 1-14, and 7-14.

Measure	Days 1-7	Days 1-14	Days 7-14
Cohen *d*	0.62	1.00	0.35
Mean changes (95% CI)	–21.2 (–18.38 to 24.06)	–29.15 (–25.98 to 32.32)	–8.15 (–6.25 to 10.06)
Mean percentage change from baseline (%)	43.0	59.1	27.8
Standardized response mean	0.82	1.20	0.66

## Discussion

### Principal Findings

The aim of this study was to undertake a psychometric evaluation of SwQoR, which comprised only negatively worded items and was completed using a mobile phone app by a population of persons undergoing day surgery. SwQoR retained the high validity, reliability, responsiveness, and clinical user friendliness of the paper-based instrument. Supporting the validity of SwQoR, all construct validity hypotheses were confirmed [22]. Reliability and responsiveness both exceeded recommended levels. Content validity has previously been demonstrated [14].

The response rate on day 1 was 86.8%, compared with 56% in Kleif et al [21] and 95% in Stark et al [20], in both of which the follow-up used the paper-based QoR questionnaire (the patients in Stark et al’s study were also required to be available for in-person or telephone follow-up). Our results suggest that using an app with a Web-based questionnaire results in higher response rates. Our response rate decreased over time, with the lowest rate of 57.5% observed on day 14. In an earlier study by the same research group, patients were asked how many postoperative days they though it would be useful to complete the instrument using the app, after using RAPP daily for 7 days postoperatively. On average, the patients considered 9 days acceptable for reporting, via an app, postoperative recovery after day surgery [14]. On day 9 in our study, the response rate was 66%, and we found no floor or ceiling effects. Only on day 14 did we find a floor effect, of 15.8%, slightly above the 15% considered to represent a floor effect [22]. However, the dwindling response rate probably reflects study response fatigue. On the other hand, there were no missing items because each item had to be responded to before submitting the daily assessment. We suggest a follow-up time of at least 10 days, but this should be further investigated.

Construct validity was strongly indicated, and SwQoR could distinguish known determinants of postoperative recovery. As stated by Terwee et al [22], construct validity is assessed by testing predefined hypotheses—for instance, concerning expected correlations between measures and expected differences in scores between “known” groups or within groups or subgroups of at least 50 participants. Without specific hypotheses, the risk of bias is high because retrospectively it is tempting to think up alternative explanations for low correlations rather than concluding that the questionnaire may not be valid [22]. Our study used well-known groups and, as hypothesized, due to the minor nature of the surgery, low correlations were found between SwQoR and duration of surgery, duration of PACU stay, type of anesthesia, and age. Stronger correlations have been reported previously for patients undergoing major surgery [10,19-21]. SwQoR discriminated between the sexes in postoperative recovery, noting poorer postoperative recovery in women than in men. Sex differences in postoperative recovery have been reported in earlier studies from Australia [10,20,25], Denmark [21], and Iran [19], although no sex differences were found in a study from Iceland [6].

We assessed discriminant validity by comparing patients with good versus poor overall health, as defined by EQ VAS scores of ≥75 or <75 mm, respectively. SwQoR clearly differentiated between patient groups, and SwQoR scores increased significantly among those with poor overall health. Discriminant validity was therefore confirmed at all 3 time points.

Overall, the test-retest reliability was excellent (*ri*=.99). We conducted a test-retest with a subset of patients (n=17) completing SwQoR twice on one of postoperative days 1 to 7 within a time frame of 2 to 30 minutes (mean 6 minutes). Our test-retest design could be a limitation in that the time frame is narrow, perhaps leading to recall bias. However, the narrow time frame ensured that the patient’s clinical condition had not changed. Earlier studies analyzing test-retest reliability in the postoperative recovery period suggest a 30-minute gap between the tests [14,20,21,26].

Regarding the SwQoR factor structure, the EFA obtained a 5-factor solution, of which 4 factors had good internal consistency [22] with an alpha range of .74 to .88. One factor, comprising 3 items, had an alpha of only .43, indicating poor correlation between the constituent items, meaning that the items could not justifiably be summarized [22]. However, all 3 items seemed to measure the same phenomena—that is, difficulties in elimination or constipation, diarrhea, and trouble urinating. The original QoR-40 items were summarized and reported across the following 5 dimensions: *emotional state*, *physical comfort*, *psychological support*, *physical independence*, and *pain* [9,10]. However, as SwQoR concentrates on individual items, not dimensions, we believe that when following up their patients, day-surgery departments should attend to specific items in evaluating and improving anesthetic and postoperative care. For example, in evaluating intravenous versus inhalation anesthesia and related postoperative differences in nausea and vomiting, follow-up and evaluation should consider “nausea, vomiting or both” values, not quality of recovery according to the *physical comfort* dimension [14]. The EFA results should therefore be treated only as a guide to organizing the items.

We assessed internal consistency using Cronbach alpha and split-half reliability; both these coefficients were high, and published recommendations for Cronbach alpha (ie, .70-.95) were satisfied [22]. However, Cronbach alpha is sensitive to the number of items, increasing with an increasing number of items [22]. These results were similar to those obtained using QoR-40 [7,10,11,19,21], QoR-15 [20], and SwQoR [14].

We measured the responsiveness of SwQoR using Cohen *d* effect size, SRM [22], and percentage change from baseline. For both the Cohen *d* effect size and SRM measures, 0.20, 0.50, and ≥0.80 are considered small, moderate, and large effect sizes, respectively [24], permitting the relative size of a change, here in global SwQoR, to be assessed. SwQoR had an effect size of 1.00 and an SRM of 1.20 between days 1 and 14. These values are equivalent to those obtained with the Swedish version of QoR [11], measuring the change in SRM between days 1 and 14 in day-surgery patients, and with QoR-40 [10,21] and QoR-15 [20], measuring the change between preoperative values and values on day 1 in patients having minor or major surgery. Our findings indicate that SwQoR has a strong ability to detect clinically important changes following minor surgery—that is, day surgery. It is an eminently suitable patient-centered, Web-based outcome measure for clinical practice and clinical trials. Responsiveness is the most important psychometric index for evaluative instruments [22]—that is, those intended to detect clinically important changes over time.

### Implications

Bowyer and Royse [8] stated that, in the future, recovery assessment would be multidimensional, be patient focused, and occur in real time at multiple clinically relevant postoperative time points. Real-time or concurrent recovery monitoring, as well as synchronous data collection, analysis, and reporting, are beneficial in any complex time-dependent system, as they minimize the delay in implementing corrective interventions to address any errors or deviations from expected norms. SwQoR can meet this need for real-time measurements. In 2008, Valderas et al recommended that future research should emphasize technological improvement, as well as the organizational and theoretical systems needed to create a care structure having patient-related outcomes as a fundamental element [27]. In our main study, 18.82% (n=338) of those assessed for eligibility (n=1796) did not have mobile phones or did not bring them to the day-surgery department [16,28], threatening the external validity. Even though 19% is low, we believe that this percentage will only decrease in the future, and adopting modern information technology to follow up patients’ postoperative recovery will be essential.

### Limitations

This study has some limitations. The study was conducted in Sweden and included only Swedish-speaking patients, so the results may not apply in other settings. We did not measure preoperative SwQoR scores, and we recruited only day-surgery patients. Furthermore, we determined neither content validity nor minimal clinically important differences for SwQoR. However, both content and cross-cultural validity, as well as agreement between positively worded and negatively worded items, have previously been evaluated [12-14].

### Conclusions

To our knowledge, this study is the first to evaluate a Web-based quality of recovery questionnaire, SwQoR, using an app installed in patients’ mobile phones. The SwQoR instrument is valid, highly reliable, highly responsive, and clinically feasible for use in systematically following up postoperative patient recovery.

## Abbreviations

ASA: American Society of Anesthesiologists
EFA: exploratory factor analysis
EQ VAS: EuroQol visual analog scale
PACU: postanesthesia care unit
QoR: Quality of Recovery
RAPP: Recovery Assessment by Phone Points
SRM: standardized response mean
SwQoR: Swedish Web version of the Quality of Recovery
VAS: visual analog scale
